# Analectic

**Published:** 1829

**Authors:** 


### MISCELLANEOUS INTELLIGENCE.

### *ANALECTIC, ANALYTICAL, AND ORIGINAL.*

### I.—Analectic.

1. *Iodine at the Hospital of St. Louis.*—Dr. Lugal, Physician to the Hospital of St. Louis, in Paris, the only establishment in which patients declared scrofulous are admitted, has adopted iodine in the cure of scrofula, with great success. His mode of administering it is two-fold:—internally, as a solution of iodine, from half a grain to a grain, in a pint or half a pint of distilled water, in which he also dissolved a certain quantity of common salt. In external applications, he used salves composed of the usual sorts of grease, with certain proportions of iodine and iodate of potassium, or the simple prot-iodate of mercury. In the space of seventeen months, M. Lugol had the opportunity of treating with this remedy 109 scrofulous patients. Of these, thirty-nine remained in the hospital at the end of the year: thirty had quitted the establishment much benefitted: thirty-six had left it perfectly cured; and there had been four on whom the treatment had proved inefficacious. M. Lugol communicated the details of his remedy, and the cures he had performed, in a memoir to the Royal Academy; and the committee to whom it was referred to inquire into the subject, reported that all the assertions of the doctor had proved exact; that the evident effect of the remedy had been established, and that M. Lugol deserved the encouragement of the Academy.—*London Athenæum.*

---

2. *Camphor Vaporized, a Remedy for Chronic Gout and Rheumatism.*—The *Journal General*, for April last, contains a memoir on this subject, by Dr. A. L. Delormel, read before the Medical Society of Paris.

The apparatus for giving the camphor vapour bath is very simple: it consists of an osier basket covered with impenetrable canvas, in which the patient may sit at his ease, with his head outside. This basket is 4 feet 6 inches in length, by 2 feet 6 inches broad and 3 feet high. Its form is oval. It separates into two equal parts, one of which has a movable lid, with an aperture in it to allow room for the head to pass through. Another aperture in front of the basket serves for the introduction of a furnace filled with live coals, which heat the interior so as to raise it to the temperature of 144 to 167 degrees of Fahrenheit (50 to 60 degrees of Reamur.) On this furnace is placed, towards the conclusion of the bath, the vessel containing the camphor, which is soon volatilized. If it be wished to augment the heat, the camphor may be allowed, without fear, to enter into combustion.

The bath is taken in the following manner. Every thing being ready, and the opening through which the head is to pass made tight, and the fire introduced, we wait until the internal air is raised to a heat of 77 degrees F. The patient, enveloped only in a flannel gown or a blanket, takes his seat, throws off his covering, and remains exposed to a steadily increasing heat during 20 minutes. The camphor is then introduced, two or three drachms sufficing for a bath. The patient remains exposed to the vaporization for 5 or 6 minutes, and afterwards resumes his gown, comes out of the bath and retires to his bed, in which the perspiration flows abundantly for two hours. The clothes of the patient are now to be entirely renewed.

The bathing may be repeated several times in the day, according to the greater or less degree of revulsion which it is desired to produce by it.

Dr. Delormel, like a man of sense, does not laud his remedy as one, adapted to all cases, and every stage of the disease. He tells us plainly that, holding in mind the powerfully stimulating effect of the vapour bath, and the imminent risk of its causing sanguineous congestions, he always directs, as a preparatory measure, one or two bleedings, according to the strength of the patient and the number of baths which he is desirous should be taken in the course of the day. For fear, also, that the digestive passages should suffer from over excitation, he takes care to have applied to the epigastrium a suitable number of leeches; and to make the patient pursue a very rigorous dietetic course at the beginning of his treatment.

The patient is directed to use habitually a decoction of dulcamara; is put progressively on a mild feculent and herbaceous regimen; and when he has taken a certain number of baths, he is put on the use of pills, of which aconitum and opium are the basis. The number of these pills must be gradually increased every day, until the summum dose is allowed: from this there is to be a regular diminution in the same ratio.

This course is often sufficient of itself to cure chronic rheumatism and gout. Sometimes we require auxiliaries—such as the application of cups—flying or permanent blisters and moxibustion.

The first case in which Dr. Delormel adopted the above practice, dates as far back as 1807. The author enumerates other instances of its success in both rheumatism and gout.

At the sitting of the Medical Society, January 2, 1829, some of the members, Messrs. Dolens and Gendrin, express their belief, that the practice is not original with Dr. Delormel. The first refers to Am. Cheze, who advocated baths of vaporized camphor in rheumatism, in an inaugural thesis of the year 1808. Dr. Gendrin said that he had employed the remedy several times himself, but without success.—*N. A. M. & S. Journal. Oct.* 1820.

3. *The use of Tartar Emetic in Large Doses recommended first in England.*—The *Medico-Chirurgical* claims for Dr. MARRYAT of Bristol the credit of anticipating the Italians in the free use of tartar emetic. Dr. MARRYAT published the last edition of his Therapeutics about 1790, and died in the year 1793. The following are his words in reference to tartarized antimony in fever, which will be found to prove that the plan adopted by RASORI, was, nearly as possible, the first tried in Italy.

"Any fever may be soon extinguished by the use of the following powders: Take of tartarized antimony five grains; white sugar (or nitre) a drachm; let them be well rubbed in a glass mortar, and be divided into six powders; one to be taken every three hours, notwithstanding the nausea the first may possibly occasion. If they bring on a diarrhœa, they should still be continued, and it will soon cease. If these are taken (which is most commonly the case), without any manifest inconvenience, let there be seven grains in the next six powders; and in the next ten. Here I beg leave to retract what I said in some former editions of this work, viz. that till sickness and vomiting was excited, this noble medicine was not to be depended on. For I have since seen many instances wherein a paper has been given every three hours (of which there have been ten grains in six powders), without the least sensible operation, either by sickness or stool, sweat or urine, and though the patients had been unremittingly delirious for more than a week, with subsultus tendinum, and all the appearance of hastening death, they have perfectly recovered without any medicinal aid, a clyster every other day excepted. I have lately seen a great many cases similar to those above, and the tartarized antimony has invariably produced the same effect."

It is but right to make us acquainted with the date of this practice of Dr. MARRYAT; and yet, we may ask whether his countrymen are not censurable for their overlooking or remaining ignorant of it so long. To Italian physicians, after all, are both English and American ones indebted for their knowledge of the impunity, not to say advantage, with which large doses of tartarized antimony are prescribed.—*N. A. M. & Sur. Journal.*

---

4. *Efficacy of sponging with Cold Water in Measles.*—In an epidemical visitation of measles in the neighbourhood of Berlin, during the autumn of 1825, Dr. THAER, a practioner of that city, determined on trying cold lotions of vinegar and water to the whole surface of the body. There were 121 sick of the disease while the epidemic lasted. Of 52 to whom lotions had not been applied, 11 died; whereas there was but one death out of 68 on whom the practice was adopted, and even in this fatal case, in which there were pulmonary tubercles, the remedy had been used contrary to the advice of the physician.

The epidemic exhibited an inflammatory character, which was considerably increased by the deplorable custom still common among the lower classes of keeping the body greatly heated under the idea of the eruption of the measles, as of all acute eruptive diseases, being thereby accelerated. A great number of those sick with measles were sufferers also from peripneumony, and some from encephalitis. The treatment before the use of the lotions consisted in keeping the air cool around the patient and employing refrigerants and leeches.

Notwithstanding this methodical course, Dr. THAER lost four of his patients—three of them had, it is true, been placed under his care several days after the invasion of the disease, and they had been kept very warm. Recourse was had to leeches in one only of the sixty-eight treated by cold ablution. They had been applied to one other before the latter treatment was commenced.

The following summary of Dr. THAER's practice and views is taken from the Revue Medicale for April.

"1. Lotions were prescribed whenever the temperature of the body of the patient was above 98° Fahrenheit, (29.5° R.) with the understanding that there were restlessness and shortness of breathing.

"The water for the lotion was used colder as the body of the patient was hotter. I was regulated in this respect by the table of FRŒLICH, which I always carry about with me, together with a small thermometer, the bulb of which I placed under the axilla of the patients.

"3. I never made use of sponging when the little patient was in a tranquil state or perspiring.

*General remarks.* 1. The children who had been thus bathed were for the most part perfectly cured in the space of eight days.

"2. The desquamation seemed to be minor in degree and more rapid in its progress after the lotions.

"3. The convalescents exposed (contrary to my orders) to the open air, which was becoming cold, did not experience on that account any inconvenience, although they had some remains of cough.

"4. When the irritation of the lungs had been of some duration, copious expectoration supervened after the use of the lotions: when the pulmonary disease was in its incipient state, it was cured without expectoration so soon as the functions of the skin had become regular.

"5. In three patients I saw the eruption come out immediately after the lotions, though prior to this there had not been the slightest evidence of it; and whenever the eruption appeared, the other symptoms were considerably moderated in violence.

The author believes that cold ablutions merit a preference over very cold air in the acute exanthemata:

1. Because in the use of lotions, we are absolutely masters of the degree of cold which it is desired to employ, so that it can be modified at any moment or season, and adapted to each individual when there are several in the same room.

2. Because water is a better conductor of caloric than air.

3. Because it is easier for a nurse or attendant to apply lotions of a reduced temperature every hour or two, than to remain constantly in a very cold room.

The better plan would doubtles be to combine the two methods, taking care always that the temperature of the room should never be below 62° Fahrenheit; and that the patient should have his habitual covering. If in despite of these precautions there should supervene much heat, with a train of other symptoms, we must have recourse to ablution, as above.

The following table is that of Dr. Frœlich, which determines at what degree of temperature the water, either for ablution or cold bathing, ought to be used, agreeably to successful experience in a great number of acute diseases:—

| Heat of the body by Fahr. Therm. | Temp. of the water by Fahr. Therm. | Duration in minutes of the Ablutions. | Baths. |
|---|---|---|---|
| 98 | 90 1-2 | 4 | ... |
| 99 1-2 | 85 | 4 | ... |
| 100 | 75 | 4 | 1-2 to 1 |
| 101 | 65 to 70 | 6 | 1 to 2 |
| 102 | 60 to 70 | 4 to 6 | 2 to 3 |
| 103 | 60 to 70 | 8 | 6 to 8 |
| 104 | 60 | ... | 3 to 4 |
| 105 | 55 | ... | 2 to 3 |
| 106 | 40 | ... | 1 to 3 |
| 107 | 40 | ... | 1 to 3 |
| 108 | 35 | ... | 3 to 4 |
| 108 1-2 | 35 | ... | 3 to 4 |
| 109 | 35 | ... | 3 to 4 |
| 110 | 35 | ... | 3 to 4 |
| 111 | 35 | ... | 3 to 4 |

*We have reduced the degrees of Reaumur in the French Journal into those of Fahrenheit.—*N. A. M. & Sur. Journal.*

5. *Subcarbonate of Iron in Tetanus.*—In the fifteenth volume, first part, of the Medico-Chirurgical Transactions, is a short paper by Dr. Elliotson, in which are detailed the histories of two cases of tetanus cured by the rust of iron. In the first, half an ounce of the medicine was prescribed with treacle and mixed with beef tea, every two hours. The symptoms declined in severity after the first two days, and ceased altogether in about twelve days. For the second, two drachms of the subcarbonate were prescribed every two hours, and the dose increased after two days to half an ounce. After eleven days of this treatment the violence of the disease having subsided, the abdomen being soft, and the trunk very little arched, the medicine was now given only every fours hours. "From this time the patient rapidly improved, had but one exacerbation in the twenty-four hours, and that at night, and gradually slighter." He was so well in a week afterwards that the medicine was discontinued. Enemata of castor oil, or of gruel, salt and oil were given at intervals. Dr. ELLIOTSON believes that in most cases of disease, doses of one to two drachms, two or three times a day, answer every purpose.—*N. A. M. & Sur. Journal.*

---

6. *Cheiloplastie (operation de).—(Operation for New Lip.)*—In consequence of an extirpation of a cancer from the lips, J. L. REINHARD, at 48 years of age, was troubled with a disgusting deformity of the mouth, and could not retain his saliva. On the 18th of June 1827, the operation for hare-lip was performed with success on the superior labium; and eight days afterwards, a similar attempt was made on the lower lip, but unsuccessfully; a second attempt was made on the 9th of July with a similar result. M. TEXTOR then determined to form a new lip from the skin of the neck. The operation was performed on the 1st of August 1827; it lasted for one hour; the flap detached from below the chin, being accurately adjusted to the vacuity to be occupied, was fixed by sutures without adhesive plaster or bandages. It is to the employment of adhesive plasters, that M. TEXTOR chiefly attributes the failure of the operations of cheiloplastic, performed by Messrs DELPECH and LALLEMAND, because they impair and interrupt nutrition in the parts compressed. The dressings were renewed the third day after the operation; on the seventh, all the sutures were removed. The cicatrix being complete on the 27th, it only remained to divide the pedicle of the flap. The deformity was greatly corrected.—*Jour. des Progress, Vol. XIV. from La Clinique.—N. A. M. & Sur. Journal.*

---

7. *Treatment of Fistulæ.*—M. LISFRANC presented to the Academy a patient who had suffered since 1826 with a fistula of the sternum; and of late, three or four new fistulæ had formed near the first. The cause was unknown; the induration and suppuration were great; the general health was much injured, and M. LISFRANC thought it would be necessary to remove the whole or part of the sternum. He first resolved, however, to attempt the improvement of the soft parts: leeches in great number were frequently applied; and when there was no increase of heat, the remaining swelling was treated by means of discutients. The fistulæ closed, and for six weeks the patient has been radically cured.

M. LISFRANC also presented a patient cured in fifteen days of a lacrymal tumour and fistula, without an operation. He employed one bleeding at the arm, leeches in great numbers applied about the ears, emollient cataplasms, emollient fumigations directed by means of a funnel into the nostrils, then resolvent fumigations, a gentle purgative, and blisters behind the ears. These measures were followed by the disappearance of the tumour. To heal the fistula more rapidly, the chloride of soda was added with advantage.

M. LISFRANC reports, that by these measures, he cured eight out of ten cases.—*Journal General, May* 1820.—*N. A. M. & S. Jour.*

8. *Recto-vaginal Fistula.*—Dr. Nicol records a case in the *Edinburgh Medical Journal* for July, in which he successfully operated for a fistulous opening between the rectum and vagina, the result of a laceration of these parts during a tedious labour. The forefinger could be passed from the vagina to the rectum, through the orifice, which was nearly two inches distant from the os externum. Considerable difficulty was experienced in keeping the vagina properly dilated during the operation, and in cutting off the edges of the fistulous opening. This was eventually overcome; the cut surfaces were retained in contact by sutures, and united completely. Dr. Nicol advises, that a considerable portion of the edges of the fistula be cut away; that the ligatures should be passed right through the rectum and so near to each other as to produce a complete mechanical closure, so that flatus even may not pass from the rectum and thus lessen the chance of adhesion. Perhaps, says Dr. N. the introduction and retention of a small gum elastic catheter in the rectum might be useful, in obviating the consequences of any such defects.—*N. A. M. & Sur. Journal.*

---

9. *Spontaneous Combustion of Both Hands.*—The following case of spontaneous combustion from the Arch. General. de Medicine for March 1829, is superior in point of authenticity to any yet recorded. A gentleman of a robust, healthy constitution and temperate habits, 24 years of age, extinguished with his hands the burning clothes of his brother, who had accidentally set fire to them with sulphur, and was immediately afterwards attacked with such acute pain in both hands as to compel him to cry out for assistance. A woman, who came to his succour, observed that both hands were surrounded by a blue flame. This at first was supposed to be occasioned by the sulphur adhering to them, and an attempt was made to extinguish the flame with cold water; but without effect. The gentleman then ran down stairs, to a cutler's shop, and plunged his hands into a quantity of mud; from this he derived very little relief. After suffering in this manner much torture for half an hour, he ran to the house of Dr. Richond-des-Brus by whom the case is related. On the way both he himself and the woman who accompanied him, observed distinctly the blue flame surrounding the hands. The physician met him at the door, and observed the hands to be red, swelled, and exhaling a kind of smoke or vapour.

He immediately directed his patient to plunge his hands into a well which was opposite, and to keep them there until he experienced relief. On his doing so, the pain abated considerably, and the flame ceased. But he had not gone more than a hundred and fifty paces homewards, when they re-appeared. On reaching his dwelling he immersed each of his hands in a bucket of water, which, as it got rapidly heated, he had repeatedly renewed. As often as he took them out of the water, he remarked a sort of unctuous matter flow along his fingers, and the blue flame re-appeared; the latter was not, however, visible, except in a situation where the light of the candle was shaded, as under the table. A young gentleman who remained in the room with him, saw the blue flame several times in the course of the night. Towards day-break sparks only were visible. During the succeeding day the pain was very severe; and large vesications, filled with a reddish serum, had formed on the fingers; in some places indeed the cuticle was entirely removed, and the cutis grayish and corroded. The vesications being opened, cerate was applied to the denuded surfaces, and the whole covered with poultices. The inflammation which followed was moderate, the suppuration healthy and in six weeks the ulcers caused by the burning were entirely healed, but the cicatrices were very distinct, and several of the nails had dropped off.

"If," observes the relater, "the flame had been perceived only immediately after the burning of his brother's clothes, it might have been supposed to have resulted from some portions of burning sulphur adhering to the skin. But it resisted the effects of repeated cold affusion, and of protracted immersion in cold water: it continued all night, and was spontaneously reproduced after having been extinguished in the water of the well. It was at first so bright as to astonish those who witnessed it, and to cause the female who first saw it to say that the hands burnt like candles. Such an explanation, therefore, is quite untenable. Although, when the patient first reached my house, I only saw a kind of smoke, yet it must be remarked, that I had a candle in my hand at the time and my servant another, so that the pale light emitted by the hands, may have been then present, but eclipsed by the superior light of the candles. If it was more visible on his return home, this might clearly have arisen from its being brighter, as well as the apartment darker. To what then should this combustion be ascribed? To hydrogen gas? But how could this gas have been generated? We might admit, as probable, that the brisk excitement occasioned by the first burning, caused the disengagement of phosphuretted hydrogen gas, which would inflame on coming in contact with the air. Thus might be explained the trivial nature of the burns. But could we also, account in this manner for the burning pain felt by the patient all the night after—for the continuance of the discharged gas, and the reproduction of the extinguished flame? Electricity does not seem to me to explain the matter any better." The case is very analogous to one which is related in the *Nov. Jour. de Medecine for Dec.* 1822.—*N. A. M. & Sur. Journal.*

---

10. *Debility of the Rectum.*—A diminution of the power in the muscles which act on the rectum in expelling the fæces, is a complaint of very common occurrence, and being attended with the symptoms of *Stricture of the Rectum,* it is frequently mistaken for it. Several cases of this kind have lately come under our notice.—*Med. Gaz.*

11. *Preservative power of Belladonna against Scarlet Fever.*—The following remarks are from the pen of Prof. Koreff, in a letter to the late M. Lænnec published in the *Bal. de Scien. Med.*

"Observation clearly proves," says he, "that the belladonna, taken for some time, either in powder or in extract, produces, especially in infants, a redness of the skin, which is sometimes transient, but at others more durable; dryness of the mouth, with a sense of heat in the throat; dilatation of the pupil; anxiety; and occasionally swelling of the sub-maxillary glands: symptoms having a great resemblance to those which accompany the eruption of scarlatina. The effects of the belladonna has also this in common with scarlatina, that neither of them produce the redness of the skin invariably, whilst the symptoms about the throat are always present. I confess to you, however, that all these analogies did not appear to me sufficiently strong to persuade me that in this plant was really to be found a preservative against scarlatina, similar to that which the cowpock affords against variola. It was not till I had received the authority of the celebrated Soemmering, who informed me that he had obtained the most satisfactory results with it when the disease raged epidemically, that I determined to employ it. This malady, accompanied by the most unfavorable symptoms, and having entirely changed its usual character, was at that time producing ravages almost as fatal as the contagious typhus. I then, for the first time, had the happiness to protect from this dreadful contagion almost all those who took the belladonna with a little perseverance, and of these there were many thousands. Since that time I have never lost sight of this discovery, which becomes the more valuable as the scarlatina has increased during the last thirty years, both in violence and extent, in many countries; and I have always found the same effects in different climates, and in epidemics of opposite characters. Many other physicians have equaly confirmed the preventive powers of this plant, and the German journals are daily filled with proofs of a benefit which, with respect to some countries, equals that of vaccination. In France, the capital and the provinces of which appear less subject to these fatal epidemics than Germany, Switzerland, the Tyrol, Poland, and the north in general, less attention has been given to this discovery, and it has been rejected, it must be said, too lightly, and without any sufficient examination, as may be seen in the article *Belladonna* in the Dictionnaire des Sciences Medicales. I only remember a single observation on this important subject, by Dr. Meglin,* who gives an account of a trial which he gave to this preservative during an epidemic of scarlatina at Colmar, and which confirms all the assertions of the German physicians. The absence of present danger is perhaps, the cause of this indifference towards a discovery, which, important in itself, might also be fruitful in results applicable to other diseases. At present, however, I shall confine myself to an account of the results which have been ascertained (by repeat-

*Nouveau Journal de Medecine, 1821.

ed observations, and by a great number of individuals placed in very different circumstances,) without incurring the reproach of having proceeded in a manner not sufficiently rigorous.

"The powder mixed with sugar, or the extract made very carefully from the juice of the recent plant, are employed after the following formulæ:—Extract of belladonna three grains, dissolved in an ounce of cinnamon water. Powder, or root of belladonna, two grains, mixed with drachms of white sugar, divided into sixty doses. From half a dose to a whole one is given to a child, from six months to two years old, four times a day; to children from three to six years old, from a dose to one and a half; to those from six to nine, two, to two and a half; to those from ten to twelve, three, to four and a half. Of the solution, a drop is given for every year of the child's age, once a day and fasting. Observation has shown that, when the epidemic is very fatal, or the intercourse with the patients very frequent and intimate, it is prudent to increase the dose a little. It has not yet been possible to determine, in a satisfactory manner, the length of time which is necessary to eradicate, by this remedy, the susceptibility to the contagion. Every thing leads us to believe that the remedy, if used during a time too short to ward off the contagion, moderates very much the malignity of the disease. We know for certain that the remedy does not permanently overcome the disposition to scarlatina; and it is necessary to resume its use on every occurrence of an epidemic. We have always observed that the most intimate communication with the sick does not produce the disease, provided the medicine has been employed eight or nine times previous to being exposed to the contagion, and continued to the period of desquamation; a circumstance important to nurses. It appears more certain to begin with rather strong doses in order to guard against the first impression of the contagion, and to diminish the quantity after a few days. No sensible effect has been observed to follow the continued use of this small quantity of belladonna. Up to the present time, neither season nor locality, nor any other circumstance, has appeared to diminish the preservative effect of this plant.

"Do not believe, my learned colleague, that these results have been too lightly deduced, or from a small number of individuals, or from epidemics of little violence. It is from entire provinces,—from cities affected with this terrible scourge,—from epidemics the most fatal, in all seasons, and in localities the most diversified,—on individuals of every age and of every condition, that observations have been made with the greatest accuracy, and have led to the above results."—*Lon. Med. Gaz.*

———

12. *Solution of Camphor.*—Dr. H. N. Preston, of Newton, recommends, in the last No. of the "Genius of Temperance," that a solution of Camphor in Lime-water be used in families, instead of the common Spirits. His mode of dissolving this gum, is to pour six ounces of lime-water on two drachms of camphor, previously rubbed down with an equal weight of quick lime. After allowing it half an hour to settle, the clean solution to be decanted.

The Doctor is of opinion that the lime can in no case where camphor is used in families, interfere with its desired operation; and the advantages of this preparation over the spirituous are, that when mixed with water, the gum will not separate; it is more economical; and, most of all, it is one important step toward the desirable banishment of alcohol from medicinal preparations. These are certainly very important objects; but we are not aware why the common Emulsion or Mixture of Camphor, which has long been in use and kept by all apothecaries, does not answer to the full all the purposes contemplated by this new preparation. The sweet almonds and sugar which enter into its composition, make it more palatable than the lime-water solution; and we should always be slow to adopt any new medicine in place of one which is well known and in common use, when the former offers no superior advantage. Physicians might well prescribe the Emulsion, in many cases where they are in the habit of directing the alcoholic solution.—*Boston Med. & Sur. Jour.*

---

13. *Fœtus affected with Fungous Hæmatodes.*—Dr. Tonnele delivered a woman of a child, which had upon its right parietal bone an enormous fungous hæmatodes. The base of this tumor originated in the osseous tissue, and perforated it like a sieve; the dura mater was healthy.—*Journal des Progres.*

---

14. *Acute Watery Effusion in the Brain occuring without Inflammation.*—In 1825 Dr. William Sherman, of London, published a small volume for the purpose of controverting "the doctrine of water in the brain being a specific disease, and to oppose the prevalent opinion of the proximate cause of the watery effusion being inflammation." In the July number (1829) of the Edinburgh Medical and Surgical Journal, this gentleman observes that since the appearance of his essay, "similar opinions seem to have been entertained, more especially as to the latter part of the above quotation, by men of extensive attainments and considerable reputation, particularly by Dr. Monro, in his book on the Morbid Anatomy of the Brain." After some other observations the following case is introduced, in which Dr. S. conceives that the effusion "was not the consequence of any preceding inflammation in the membranes of the brain, but arose as an accidental occurrence in a weakly and scrofulous constitution; predisposed, therefore, to a great degree of irritability of those membranes."

A child three years old, of a weakly constitution, and scrofulous appearance, was brought, November 6th, to the West London Infirmary, with syptoms of hydrocephalus. His head was constantly in a rolling motion, accompanied with convulsive twitchings in his limbs. His countenance had an idiotic expression, and he was in capable of recognizing his mother or surrounding objects. The pupils were greatly dilated; the child appeared unable to see, a near approach of the hand of a bystander to the eyes, not producing any motion of the eyelids. The pupils, however, slightly contracted on the approach of the light of a candle, but not otherwise. Bowels somewhat constipated; but there was neither hardness nor tension of the belly,—pulse nearly natural. The child had not spoken for many days. The mother stated, that two weeks previously he was attacked with febrile symptoms, accompanied by purging. These continued for a week, when she discovered in the morning, that the child was unconscious of her presence, and incapable of seeing, and exhibited all the other symptoms now present. A blister was applied to the right side of the abdomen, and a grain of calomel directed every two hours. November 9. Blister had drawn and discharged freely; twelve grains of calomel had been taken, but at rather longer intervals than prescribed. Bowels freely opened, urine copious. The child was now conscious of the mother's presence, and of surrounding objects; can see perfectly. The rolling motion of the head, and convulsive twitches have ceased. Ten drops of sulphuric acid and of vinegar of squills were directed every four hours, and four grains of rhubarb and magnesia, with one of calomel, every night. By the 20th of the month all symptoms removed. The medicines were continued for a short time longer, with the addition at night of three grains of ferrum tartarisatum.

In relation to this case, Dr. S. remarks, that he "can see no evidence of inflammation of the brain or of its membranes having taken place during any time of the progress of the disease; no prominent symptoms of the head being particularly affected, manifested themselves in the space of the first week; and the sudden accession of those denoting effusion of fluid would seem to be the *effect* of that state of the system, whatever it was, to which the febrile symptoms were owing. If it be maintained," he adds, "that these febrile symptoms themselves were nothing more than symptoms of inflammation of the brain, to viz. that they constituted hydrocephalus intermus in its first stage, it will be necessary to explain why this inflammatory disease should have terminated in perfect recovery, without the counteracting means, whilst almost all severe cases of a similar kind, where large bleedings and antiphlogistic measures have been employed, terminate fatally."

We present to our readers the outlines of the foregoing case, as we consider it to be one highly interesting in many points of view. We must say, however, that we cannot receive it as a proof of the non-inflammatory character of hydrocephalus. There is in fact no positive evidence that watery effusion had taken place in the brain of this patient; on the contrary, there is every reason to believe that it had not. Dropsy of the brain we know to be a disease very generally fatal, whereas in the case before us all the symptoms attributed to the existence of effusion within the brain disappeared in a few days, under a treatment certainly not the most active. We have seen cases in which phenomena, very similar to those which occurred in Dr. S.'s patient, though probably not to the same extent, were very evidently connected with gastric irritation, ceasing almost suddenly upon the expulsion of undigestible substances or worms from the stomach. In these instances it is very certain that water did not exist in the brain.—*N. A. Med. & Sur. Jour.*

---

15. *Tartar Emetic in Large Doses.*—We take the following summary in Dr. Bayle's work, *Bibliotheque de Therapeutique*, from the *Journal General* for June last.

1. Tartar emetic administered internally, in disease, in doses of from 8 grains to a scruple or drachm, and even several drachms during the day, is not a poison; it is not even followed by bad effects, except in a small number of cases, in which manifest counter-indications were present.

2. Whether borne or not by the sick, it does not produce in them inflammation of the gastric intestinal mucous surface. When evidences of this phlegmasia exist, such as redness of the tongue, pain of the epigastrium, diarrhœa, we often find them disappear under its use (Laennec, Delormel, Meriadec-Laennec, Lagarde, Fontanelles); when the patient dies we commonly find the digestive tube exempt from alteration, and the internal membrane pale, or slightly injected (Meriadec-Laennec, Strambio, &c.)

3. Tartar emetic in large doses is a powerful remedy in *peripneumony*. It is very useful either as an auxiliary to bleeding, or as the sole curative means, when sanguineous emissions have not arrested the progress of the disease, or when we deem it improper to have recourse to them.

M. Peschier cured all his patients with the exception of one, without bleeding, and entirely with the tartar emetic. M. Wolf has cured *ten*, all that he treated; M. Palais, *one*; M. Prato, *two*; M. Rasori, *fifty-two* out of *sixty-one*, in his *civil clinic*, and *fifteen*, being the whole number, in his military clinic.

As to the pneumonic cases which were subjected to both blood-letting and the use of the tartar emetic, we learn that Rasori cured in his civil clinic *four hundred and forty-four* out of *six hundred and two*, which makes a mortality of twenty-two out of a hundred. In his military clinic he cured a *hundred and forty-nine* out of a *hundred and seventy-five*—deaths *twenty-six*—mortality fourteen per cent. M. Laennec out of *fifty-seven* cases cured *fifty-five*—deaths *two*, which is equivalent to rather less than one in twenty-eight. M. A. Laennec cured *thirty-seven* patients out of *forty*—deaths three, or one in thirteen.

The greater number of these persons took the tartar emetic without its producing vomiting, or at least only shortly after its first administration. In others it was not retained on the stomach, although this circumstance was not always an obstacle to the cure.

4. *Articular Rheumatism* is, next to pneumonia, the inflammatory affection in which the antimony in full doses has been the most efficacious. Among a great number of cases treated by M. LAENNEC, this professor found that the mean period of the disease under this treatment was seven or eight days. Of thirteen cases collected from his clinic, the tartrate of antimony and potass was evidently useful in *eight*; it was without effect in *two*; hurtful in *one;* and of doubtful efficacy in *two* (MERIADEC-LAENNEC). Out of five cases of acute articular rheumatism, M. HONORE cured *four* (LAGARDE). Of fifteen cases cited by M. DELORMEL, *thirteen* were followed by a cure. The *Observer* of *Naples* contains *six* other cases of cure, *two* of which have been published by Dr. SPADAFORA.

5. Tartar emetic in large doses has been tried in many other affections, but on too small a number of patients to inspire us with an entire confidence in the success of the practice.

M. LAENNEC has cured by this means, an *arachnitis*, three cases of *acute hydrocephalus*, a *phlebitis*, three cases of *chorea*, and two *anginas*. M. A. LAENNEC has succeeded in two cases of *idiopathic* tetanus. M. RECAMIER in four cases of acute pulmonary catarrh. M. FONTANELLE in one of *jaundice*.

6. In other diseases in which this mode of treatment has been tried, there are many cases of its failure, and some of its having been injurious. M. LAENNEC has remarked that the tartrate of antimony and potass produced a prompt reduction of the inflammatory organs in pleurisy, but that it did not hasten the absorption of the effusion which is the consequence. Out of eleven cases of apoplexy, six have been cured; but as the professor made use at the same time of blood-letting, it is uncertain whether the tartar emetic contributed to the cure (MERIADEC-LAENNNEC). In a case of rheumatism, and another of gout, it was evidently hurtful (MERIADEC-LAENNEC). Its employment in *insanity*, accompanied with *semi-paralysis*, was not in general attended with any success.—*N. A. M. & S. Jour.*

---

16. *Partial Palsy cured by Strychnia, locally applied.*—The subject of this case was an habitual drunkard, aged 36 years. He had lost the power of the left fore arm and hand ten days previously. The sensation of the parts was perfect, but the mobility much impaired. There was no head ach. Over a vesicated surface on the back of the fore arm, one eighth of a grain of strychnia was sprinkled. The dose of the medicine was increased by doubling the quantity every day until it amounted to one grain, after which a fourth of a grain was to be added. The improvement was manifest from the second week, and the patient, without having experienced any uneasy symptom, was dismissed cured at the end of five weeks from the commencement of the treatment.

In another patient, affected with paralysis of the flexor muscles, and diminished sensation of the right leg, a cure was effected in the course of six weeks.—*N. A. M. & S. Jour.*

17. *Inflammation of the Sinuses of the Brain.*—During the present year M. Tonnele read before the Royal Academy of Medicine a memoir upon the diseases of the sinuses of the dura mater. We shall notice on the present occasion only so much of the memoir as relates to inflammation of these vessels. The fact that the sinuses of the brain were, like the veins, liable to inflammation, and to the formation of false membranes in their interior, was established in a paper published by M. Ribes in 1825.* Of these inflammations M. Tonnele reports several cases, which he arranges according as the pseudo-membranous concretions occur with or without suppuration. Of the first we notice particularly the three following. 1. An infant two years old, after the disappearance of an eruption of the scalp, was affected with several symptoms of cerebral disease; as peevishness, somnolency, contraction of the limbs, dilatation and insensibility of the pupils, &c. After death, pseudo-membranous concretions, with suppuration, were discovered to exist in the superior longitudinal sinus and in the veins of the pia mater; there was also an ecchymosis in the tissue of the latter membrane and a superficial softening of the brain. According to M. T. this case presents an instance of inflammation of the internal membrane of the sinus, producing false membranes, and finally ramollissement of the brain. 2. A female child two yeats old, affected with scrofulous ophthalmia, was suddenly seized with convulsive movements of the muscles, and other indications of cerebral disease: these were shortly replaced by syptoms of adynamia, which caused the death of the patient in a few days. Upon the right side of the occiput there was discovered an ulcer, the existence of which had been overlooked. *Sectio cadaveris.* The right laternal sinus was twice the ordinary size, and filled with thick, fetid pus, containing small solid concretions and numerous clots of blood; the internal surface of the sinus was covered with a thick false membrane; the dura mater, where it forms the sinus, as well as the corresponding parts of the cerebellum, were covered with a layer of pus, and the nervous substance beneath this pus was softened. M. T. conceives that in this case the ulcer at the occiput was the cause of the inflammation of the sinus, in consequence of the pus having been taken up by the absorbents and carried into the former. With respect to the syptoms under which the patient laboured during life; he refers the convulsive movements and other symptoms of cerebral disturbance to congestion and inflammation of the brain and of its meninges; while he ascribes the symptoms of adynamia to a poisoning of the blood by the absorbed pus. 3. An infant two years old, for a long time in a weakly condition, died suddenly as if from suffocation. On examining the body, it was found that a quantity of blood had escaped under the pericranium through the sagittal suture; the superior longitudinal sinus, to the extent of two-thirds anteriorly, was distended by a large coagulum evidently of recent formation; throughout its posterior third it was lined with false membranes mixed with a cream-like fluid. The veins which terminated in the sinus were very much distended. In the centre of the right hemisphere of the brain was discovered a large cavity filled with blood, to which the sudden death of the child is to be attributed. In those cases in which there exist in the sinuses false membranes without any collection of pus, the effects are the same, viz. impediment to the circulation of the blood; effusion of the latter on the surface of the brain; infiltrations of blood in the arachnoid and in the cellular tissue beneath this membrane; during life symptoms of nervous and cerebral disease; in some cases sudden death.

The causes by which inflammation of the sinuses of the brain is produced, M. T. observes, it is difficult to determine. In the majority of the cases which he has observed it occurred in patients labouring under tinea capitis, or in whom there was also a development of false membranes in several other parts of the body.—*N. A. Med. & S. Jour.*

---

18. *On the detection of Corrosive Sublimate. By* M. Orfila.—In an article, contained in the June number of the Journal de Chimie Medicale, M. Orfila comments upon the method proposed by Mr. James Smithson, for detecting minute portions of corrosive sublimate; which consists in plunging into the suspected liquid a small electrical pile, formed of a gold ring, wound round in spiral by a rolled piece of tin-foil, and then adding a few drops of muriatic acid. Mr. Smithson alleges, that sooner or later, if the liquid contain corrosive sublimate, the ring will become whitened by a precipitation of the metallic mecury.

M. Orfila admits that corrosive sublimate may be detected in this manner; but asks whether this mode of proceeding may not lead to error, by giving very similar results, when employed for solutions which contain not a particle of mercury. M. Orfila thinks it may; and adduces in support of his opinion, the following facts:

A sudorific syrup, called a *regenerator of the blood*, having been submitted to his examination, he tested it by the usual reagents for detecting minute portions of mercury, without success; and then had recourse to Mr. Smithson's method. At the end of two hours the gold exhibited white spots, resembling those which might be expected to result from a weak solution of a mercurial salt; and upon exposure to heat, it resumed its yellow colour, just as happens to gold whitened by mercury. M. Orfila was hence strongly inclined to believe that the syrup contained a mercurial preparation, and nevertheless it contained none. To render the matter certain that the whitening of the gold did not depend upon a thin coating of metallic mercury, M. Orfila prepared the syrup himself, without using any mercurial preparation, and, after acidulating it by a few drops of muriatic acid, plunged into it the minute electrical pile. At the end of twenty-four hours, the gold was whitened, and when subjected to the fire was similarly affected, as it would have been, had it been covered by a minute film of mercury.

M. Orfila undertook to determine the cause of this appearance; an investigation of the more importance, as calculated to guard physicians and pharmaceutists from committing errors, when charged with making reports on subjects connected with the administration of justice. The explanation which occurred to him was, that a small portion of muriate of tin was formed, and that this was decomposed by the elementary pile, and the metallic tin deposited on the gold which it whitened; and that when the whitened gold was exposed to heat, the tin combined with the gold, and formed an alloy containing so little tin as not to impair the colour nor general appearance of the gold.

M. Orfila made several experiments, and satisfied himself that the explanation above mentioned was correct. He found, however, that if plates of gold and tin were *separately* suspended in a weak solution of muriatic acid, the whitening of the gold did not take place. Hence it occurred to him, that if Mr. Smithson's plan, thus modified, would detect mercury by the whitening of the gold, it would be free from all ambiguity from the danger of the whitening being produced by the tin. The experiment being thus modified, resulted in giving to the gold a slight and loosely adhering coating of calomel, on the metals being separately suspended in a weak solution of corrosive sublimate. Thus it was shown, that to obtain the result of a coating of metallic mercury on the gold, the minute pile is necessary.

M. Orfila next examines the following question: is it practicable to distinguish whether the gold of the little pile is spotted white by mercury or by tin? He found that the gold when whitened by the tin might be restored to its natural colour in a short time, by the application of concentrated and pure muriatic acid; but when whitened by mercury and similarly treated, the whitened portions resisted the action of the acid, even for twenty-four hours. Hence it is obvious that muriatic acid furnishes a means of distinguishing whether the gold be whitened by mercury or tin. M. Orfila, however, prefers that the discrimination should be made by subjecting the whitened gold to heat at the bottom of a small glass tube, drawn by means of a lamp to a capillary orifice. If the white colour arises from mercury, it will be volatilized, and condensed in the small end of the tube. If however, it be from tin, no such effect will take place.

M. Orfila concludes his paper by remarking, that Mr. Smithson's little apparatus, when employed with the precautions necessary to determine the nature of the whitening metal, "is without contradiction, the most delicate reagent that can be employed for the detection of minute traces of a mercurial salt."

[We would suggest, that the small pile of Mr. Smithson might be formed by combinations of gold, severally with copper, or iron. If these combinations would succeed in precipitating mercury, they would be free from the ambiguity attendant on the use of tin.—Ed.]

*N. A. Med. & Sur. Journal.*

19. *New organic Alkalies discovered in Cinchona Bark.*—We derive the following interesting article from the *Journal des Progres*, Vol. III. for 1829. Dr. SERTURNER, chemist, of Hamelem, remarked, during the prevalence of an epidemic intermittent fever in 1828, that the quinia was far from being a certain specific against this disease, even when given in doses of six or eight grains, conjoined with acids. It stopped the paroxysm, but not in a permanent manner; for there were many relapses, which made it necessary to resort to the bark in substance, which, administered in large doses, in conjunction with acids, rendered the relapses much less frequent. Dr. SERTURNER likewise ascertained, what has been observed by other practitioners, that quinia cannot be substituted for cinchona, as a tonic. He was therefore, induced to undertake new analytical researches on the different kinds of cinchona bark, with the view of ascertaining the cause of this difference. The details of his experiments, he reserves for future publication, communicating at present his principal results, which are as follows:—

The precipitates obtained by treating the acidulous extracts of cinchona bark by alkalies, comprise, besides quinia and cinchonia, certain additional organic alkalies, which may be considered as modifications of the former.

These new organic alkalies, and especially the principal one, which Dr. S. calls chinioidia (*chinioidine*) are intimately united with a sub-acid resinous substance, which, if not hurtful, is at least not beneficial, and which is very difficult to separate. Its separation can be effected completely, only by the vegeto-animal charcoal, which is obtained in the preparation of safranic acid, discovered by M. LIEBIG, after having dissolved in strong sulphuric acid, (diluted with three or four times its weight of water) the impure alkaline substance, which remains after the sulphate of quinia has been separated by crystallization, the solution is to be decolorized by means of a mixture of the vegeto-animal charcoal, above mentioned, with ordinary animal charcoal. But, before conducting this decoloration, it is best to treat the solution with alcohol, in order to separate the earthy salts.

The new alkali exists in the cinchona barks, associated with quinia and cinchonia.

*Properties of chinioidia.* It resembles the other alkalies of the cinchona bark, in its insolubility in water, its colour and taste. But it is distinguished from them by its activity, and its greater capacity of saturation. Its alkaline reaction, and its intimate combination with an extractive matter, which is probably an acid, are characters not less striking. Its salts, when freed from extractive, are affected by heat and liquids, after the manner of balsams; being viscid and fusible like the latter, although they contain frequently, to all appearance, their acids in a dry state. As a medicine, chinioidia is one of the most precious agents of the materia medica. It is not merely a better febrifuge than quinia, and even than the bark in substance; but it possesses many other therapeutic properties, which, admitting that they exist in the bark itself, are not to be found in quinia. It was prescribed by Dr. Serturner [in saline combination?] in the dose of two grains, three times a day, with the direction to swallow a little vinegar after each dose with the view of saturating the gastric juice, which is sometimes alkaline in fevers, and which by acting on the salt, sets free the chinioidia, and thereby renders the medicine insert, in consequence of the insolubility, of the new alkali, when uncombined. In all the cases, treated by the new remedy, the fever was cut short without relapse, and in every instance, the concomitant symptoms, such as the paleness of face, loss of appetite, œdema of the legs, &c. disappeared in as horter time than is usually the case. The medicine failed only in a single instance. The quantity necessary to effect a cure was generally from twelve to twenty-four grains.—*N. A. M. & Sur. Journal.*

20. *Ophthalmia Neonatorum.*—Under this title, Mr. Wishart of Edinburgh, has published an able article on the purulent ophthalmia of infants, of which we shall endeavor to present our readers a brief analysis.

The disease usually commences in from three days to four weeks after birth. The eyelids are first observed to be frequently glued together, so that the child has considerable difficulty in separating them, as is evident from the action of the muscles in opening the eyes. They are however generally open in a moderate light, and still more so if the room is darkened. On farther examination, the conjunctiva of the ball is found clear, but that of the lids is observed to be red, puckered, and covered with a white, mild, thick slime. The difficulty of opening the lids, the intolerance of light, the swelling and redness of the conjunctiva, gradually increase as the disease advances. At length the lids remain constantly shut, and any attempt to separate them occasions great pain to the patient. This can only be effected during sleep, by moistening their edges with lukewarm water. On making the separation, a copious discharge takes place of thick mucus, varying in color from white to a greenish yellow, and often mixed with streaks of blood. The quantity of this is in proportion to the violence of the inflammation, and the length of time that the lids have been allowed to remain undisturbed. Still, however, the redness is confined to the conjunctiva of the lid, or if the ball is inflamed the injected vessels are not so numerous but that they apppear perfectly distinct.

At this period, an attempt to open the eye is not unfrequently followed by eversion of the lid. This eversion must be carefully replaced; otherwise the lining membrane thus exposed to the air becomes more red and swollen, and acquires the appearance and character of inverted *rectum.* It will sometimes happen that the lid can not be reduced, and the eversion is permanent. This state of things is usually followed by the gradual failure of the patient.

An occurrence not uncommon at this stage of the disease, is a greater or less hemorrhage from the lids. This, though alarming, is a favorable occurrence; for by this bleeding turgid vessels are emptied, and the inflammation diminished. If, however, the progress of the disease is not checked, the symptoms which ensue are more formidable. In consequence of the swelling of the lids, the edges of the tarsus are contracted, and the matter secreted can no longer escape. Rendered acrid by confinement, it increases the inflammation of the eyeball, an ulcer forms on the cornea, which becomes gradually deeper till the membrane is perforated, and the consequences which ensue terminate only with the entire destruction of the organ.

Among the causes of this disease, the more obvious are those which produce other complaints among the poorer classes,—namely, exposure to cold or dampness, or to a strong light, as in too suddenly admitting the glare of sunshine, or in dressing the child before a large fire. A much more frequent cause, however, than is generally suspected, is the existence of morbid uterine discharge, whether venerable or otherwise, in the parent at the period of delivery. More than two-thirds of the infants affected, are born of mothers laboring under leucorrhœa.

The complaint, when treated in the best way, seldom continues less than three weeks, and if neglected or unskilfully managed, may be protracted to ten or twelve. The morbid changes most frequently left by it, are the ectropium or eversion already mentioned, and opacity of a portion of the cornea. Neither of these occur except in the worst cases, and are for the most part owing to injudicious treatment.

With regard to the prevention of this complaint, two precautionary measures are suggested by the considerations already mentioned, —one, to remove if possible any morbid discharge existing in the parent previous to delivery, and the other, to wash the eyes of the infant carefully immediately after birth.

The disease, as already stated, seems to have a certain regular course, and a violent or sudden interruption of its progress would be dangerous to the patient. The following mode of treatment, however, is safe and effectual. If the case is seen within a week from its commencement, which is as soon as generally happens, the purulent discharge is to be carefully washed away with warm water, and the following lotion ordered:—

R. Sulph. Zinci ℈i.

Aquæfont ℥x. Solve et adde

Liq. Subac. Pl. ʒi.

Tinct. Camph. ʒi.—ʒij. M. et cola.

This is to be carefully injected three times in a day with a fine pointed ivory syringe; at first diluted a little with hot water, so as to be rendered tepid, in which state it answers better, especially in cold weather. If the discharge be very great, the intervals of its application must be abridged. In the state above mentioned, it generally produces pain, which continues from five to ten minutes; if the child cry longer than this, it ought to be diluted. In the mean time, the eyes are to be frequently washed with warm water, and at night a small quantity of the ung. ox. zin. is to be inserted between the lids. Leeches or scarification may be added to the treatment, if necessary.

At the end of two weeks, if the cure goes on well, the inflammation will have diminished, and the discharge will acquire a watery appearance. At this period, the ung. hyd. ox. rub. may be substituted for that above mentioned, and the lotion altered to the following:—

R. Mur. Hyd. gr. i.

Aq. Ros. ℥ vi. M. et adde

Vin. Op. ʒ iss. M. f. coll.

At the end of a month, it generally becomes unnecessary to use the syringe, and the collyrium may then be continued occasionally by dropping a portion on the inner angle of the eye, and allowing it to pass over the surface.

The most usual sequelæ of purulent ophthalmia, as above mentioned, are opacity of the cornea and ectropium. The former complaint is removed in young infants without difficulty. The best treatment consists in the use of the ung. hyd. prec. at night, and the vin. opii. more or less diluted, in the morning. The eversion of the eyelid, when recent, is easily reduced, and rarely becomes permanent or irreducible, except in consequence of neglect. When it is so, however, the conjunctiva must be treated with the ung. hyd. prec., or some mild caustic, until the swelling is diminished, when an attempt must again be made to accomplish its reduction. The sooner this is effected, and the changed surface is withdrawn from the action of the air, the sooner will it return to its healthy state.

*Bos. Med. & Sur. Journal.*

---

21. *Influence of the Age of the Parents on the Sex of their Offspring.*—The interesting but mysterious subject of conception, and the laws, if any exist, which regulate the sex of the offspring, has received, of late years, an unusual share of the attention of the faculty. The following are the result of some researches on this subject, by Professor Hoffnacker, of Inspruck, published in the Inspruck Med. Chir. Zeitung.

1. In marriages where the mother is older than the father, the average number of male to that of female births is 90. 6: 100.

2. Both parents being of the same age, the proportion of boys to girls is 92: 100.

3. The father being from three to six years older than the mother, the number of male to that of female children is 103.4: 100.

4. Where the father is from six to nine years older than the mother, the proportion is 124.7 boys to 100 girls.

5. The age of the father being from nine to twelve more than that of the mother, the proportion is 143.7 : 100.

6. Where the age of the father is eighteen years and more above that of the mother, the proportion of male to female births is 200 : 100.

7. Young men, from twenty-four to thirty-six, produce with young women, from fourteen to twenty-six, 116.6 boys to 100 girls.

8. If men between the age of twenty-four and thirty six, are married to females between thirty-six and forty-six, the proportion of male to female children is 95.4 : 100.

9. Middle-aged men, from thirty-six to forty-eight years, being married to young females, the proportion of their male and female children is 176.9 : 100.

10. Middle-aged men, and middle-aged women, produce 114.3 male to 100 female children.

11. Middle-aged men, being married to women of a more advanced age, the proportion of male to female children is 109.2 : 100.

12. Old men and middle-aged women produce 190 male to 100 female children.

13. If husband and wife are both equally advanced in years, the proportion of their male and female children is 164.3 : 100.

*Bos. Med. & Sur. Journal.*

---

22. *Mode of suspending the Secretion of Milk.*—M. Ranque, chief physician to the Hotel Dieu of Orleans, employs with success, to diminish the sensibility of the mammary gland, upon which the secretion of milk depends, frictions morning and evening upon the breast, with the following liniment :—R. Laurel water ℥ij.; sulphuric ether ℥i. ; extract of belladonna ℈ij. He prescribes at the same time rigid diet and sudorific drinks.

M. R., it is said, employs this liniment with success in engorgements of the testicles, after using antiphlogistics.—*Journal des Progres.—Bos. Med. & Sur. Jour.*

---

23. *Treatment of Imperforate Rectum*.*—Imperforate rectum, or imperforate anus, is not only a dangerous, but unhappily a much too frequent malformation, and one in which surgery alone can be of service. At page 515 of the last number of this Journal, we related the particulars of an interesting case which occurred in Paris to M. Piedagnel, and we shall now avert briefly to one or two others that have since been recorded.

In February of the present year, an infant three days old, was brought to M. Dupuytren at the Hotel Dieu, with the anus well formed, but opening into a culde-sac, which admitted the extremity

---

*Journal Hebdomadaire, No. 23.

of the little finger. The cul-de-sac appeared to be in contact at its apex with the rectum dilated by meconium, and pressure on hypogastrium rendered this sensation communicated to the finger, still more distinct. The accoucheur had made some unsuccessful attempts to open the intestine with a lancet. The belly was tense, tympanitic, and painful—there was frequent vomiting—and, under these alarming circumstances, M. Dupuytren was afraid to adopt the method of Durat, and establish an artificial anus in the left groin. Instead of this procedure M. D. introduced a trocar in the natural direction of the rectum, but only gave issue to a little gas, and the poor infant died in the course of the day.

At the same time that this case occurred another child five days old, was brought for the inspection of the same distinguished surgeon. In this there was no cul-de-sac, and the anus was only slightly marked—there had been some vomiting—belly slightly tense and painful. In this, as in the former instance, a lancet had been introduced without success. M. Dupuytren wished to open into the descending colon in the left groin, but the parents refused their consent. A puncture was made with a trocar in the usual situation of the rectum, but nothing was discharged, and the infant was consigned to those who had the care of it.

The foregoing are melancholy instances of the fatal effects of imperforate rectum, and we turn with pleasure to one of a more pleasing cast, recorded by Mr. Miller in the January number of the Edinburgh Journal. We make no apology for noticing it shortly in this place, as it gives an additional interest to the subject.

In 1821, Mr. Miller attended Mrs. —— in her confinement, and assisted to bring into the world a male infant, who at first was supposed to be perfectly formed, but was soon discovered to want the natural outlet of the intestines. No trace of an anus existed, but the meconium was passed by the urethra, shewing that the rectum opened into the bladder. Mr. Miller made an incision with the scalpel in the situation of the anus, an inch and upwards in length and very deep. He then plunged a trocar in the supposed direction of the gut, and fortunately for the child, and to its great relief, meconium issued through the canula at the second attempt. When the canula was removed, pieces of sponge-tent were introduced, but they could not be borne for any time, and injections of warm gruel were employed.—In spite of every care, however, the aperture narrowed so much during cicatrixation, that it was necessary to enlarge it ten times by a histoury in the course of eight months. The child grew, but with its growth contracted an unfortunate penchant for coal-cinders, which he constantly eat, and which, almost as constantly stuck in the passage, and required frequent operations to remove them. In one of these, the bladder was wounded, and an urinary fistula in the rectum was the consequence. He possessed the power over the sphincter ani, but required the frequent use of laxatives and glysters; in administering which about three years ago the mother perceived that some substance opposed the introduction of the pipe. Little however, was said about it, till at length the obstruction of the bowels was complete, and Mr. Miller on examination found a calculous concretion of very large size, blocking up the passage, and felt with a probe through the orifice of the anus.

"As the anus would only admit a goose quill, I enlarged it sufficiently to allow of a full examination with my finger, when finding the stone almost to fill the hollow of the sacrum, it was useless to think of extracting it entire, as I felt satisfied that the bones alone would absolutely frustrate every such attempt. Drilling it to pieces by boring in different directions, at once struck me as the only method of proceeding, and I unwillingly left the little sufferer for another day, that I might provide the appropriate instruments. Having procured, from an ingenious mechanic, a drilling apparatus to be wrought with a bow and string, (resembling a common hand-drill) I had a few drill-bits formed of a conical shape and of various sizes, to make a bore of from 1-4th to 5-8ths of an inch in diameter. The greatest difficulty appeared to be the properly fixing the stone when in the act of drilling. The impossibility of introducing the usual forceps to grasp such a stone, suggested to me the propriety of having a pair constructed on purpose, with separate blades, which might be fixed with a screw when applied; and such a pair being promptly prepared by Mr. Marshall, cutler, and having obtained the valuable assistance of my friend, Mr. James Miller, surgeon at Perth, I enlarged the anus in the direction of the sacrum as far as the coccyx would allow, and in the direction of the pubes, as far as the safety of the urethra would permit. The separate blades of the forceps were now cautiously introduced, and secured by turning the screw. I then applied the drilling apparatus, while my friend, Mr. Miller, held the stone firmly at the orifice betwixt the blades of the forceps, using every precaution to avoid injury. The drills were steadily and perseveringly applied, first of the small and then of the larger size, until considerable openings were made in the stone in various directions. A pair of strong polypus forceps was now thrust into the openings, and the blades being forcibly separated by extending the handles, the calculus gave way, and was separated into three pieces, each of which had in its turn to be drilled and broken in the same manner before it could be extracted. The whole operation occupied not less than two hours and three-quarters; and it was truly surprising to see with how much fortitude the little fellow bore such protracted suffering."

No bad symptoms of any kind followed. The patient had perfect power over the sphincter ani—and Mr. Miller believes that, under the use of the elastic gum catheter, the vesico-rectal fistula will heal. The calculus was at least the size of a turkey's egg, but the *practical* portion of the case being what we want, we need not stop to describe its chemical composition. The proceeding adopted does much credit to Mr. Miller's ingenuity, and may serve as a precedent in a similar case. It is obvious that the *lithontripteur* of M. Civiale, or still more the improvement upon it carried into effect by Dr. Frecchioli, of Florence, and described in a recent number of this Journal, would be infinately preferable to a comparatively clumsey and unhandy complication of instruments.—*Med. Chir. Review.*

---

24. *Supposed Hernia of the Lung, in consequence of Tight Stays.**—A young woman, 18 years of age, of good constitution, and who had menstruated regularly, presented herself to M. Breschet with a tumour in the right side of the neck. It rose from behind the clavicle to as high as the level of the thyroid cartilage; was broader at its base than at its apex; was of variable size, but at its maximum about the dimensions of the fist; on compression, felt elastic and soft; disappeared entirely if the pressure was made from above downwards and inwards, but again returned on withdrawing the pressure, especially if the stays were laced at the time; was perfectly indolent, and presented no pulsation. On applying the stethoscope to the tumour, the respiratory murmur was distinctly heard, and no doubt remained, from this and the preceding symptoms, that the enlargement was produced by the summit of the lung protruded from the cavity of the chest; in other words, by a hernia of that organ. The disease was produced by the habit in which the young woman, like most other females of her age, had indulged, of wearing the stays exceedingly tight laced. When she did this she always experienced a difficulty of respiration, and the volume of the tumour increased. M. Breschet directed this patient to leave off her stays, and proposed to apply compression to the surface of the hernia.—*Med. Chir. Review.*

